# Novel analysis of proton-γ coincidences for the β-delayed proton emission technique: the case of $^{46}$Mn

**DOI:** 10.1140/epja/s10050-026-01952-y

**Published:** 2026-09-03

**Authors:** D. Godos, L. Acosta, P. Ascher, B. Blank, J. Giovinazzo, F. de Oliveira Santos, C. Fougères, A. M. Sánchez-Benítez, N. Adimi, A. Algora, M. Aouadi, N. Benouaret, C. Borcea, Y. Fujita, M. Gerbaux, T. Goigoux, S. Grévy, G. F. Grinyer, O. Kamalou, T. Kurtukian Nieto, A. T. Laffoley, C. Magron, D. Nishimura, S. E. A. Orrigo, T. Roger, B. Rubio, J.-C. Thomas, M. Vandebrouck

**Affiliations:** 1 Instituto de Estructura de la Materia, CSIC, C. de Serrano, 121, 28006 Madrid, Spain; 2 Instituto de Física, Universidad Nacional Autónoma de México, Circuito de la Investigación Científica, Ciudad Universitaria, 04510 Mexico City, CDMX Mexico; 3 Dept. Ciencias Integradas, Facultad de Ciencias Experimentales, Centro de Estudios Avanzados en Física, Matemática y Computación, Unidad Asociada GIFMAN, CSIC-UHU, Universidad de Huelva, 21071 Huelva, Spain; 4 Laboratoire de Physique des 2 infinis de Bordeaux, Site du Haut Vigneau, 19 chemin du Solarium, 33170 Gradignan, France; 5 Grand Accélérateur National d’Ions Lourds, CEA/DRF-CNRS/IN2P3, B.P. 55027, 14076 Caen Cedex, France; 6 CEA, DAM, DIF, 91297 Arpajon, France; 7 Laboratoire Matière en Conditions Extrêmes (LMCE), Université Paris-Saclay, CEA, 91680 Bruyères-le-Châtel, France; 8 SNIRM, Faculty of Physics, University of Sciences and Technology Houari Boumediene USTHB, B.P. 32, El Alia, 16111 Bab Ezzouar, Algeria; 9 Instituto de Física Corpuscular (IFIC), CSIC-Universitat de València, C. Catedrático José Beltrán, 2, 46980 Paterna, València Spain; 10 IFIN-HH, P.O. Box MG-6, 76900 Bucharest-Magurele, Romania; 11 Department of Physics, Osaka University, Toyonaka, 560-0043 Japan; 12 Department of Physics, University of Regina, Regina, SK S4S 0A2 Canada; 13 Department of Natural Sciences, Tokyo City University, Tamazutsumi, Setagaya City, 158-8557 Japan; 14 Université Paris-Saclay-CEA-IRFU, 91191 Gif-sur-Yvette, France

## Abstract

In the interest of exploring resonant contributions to the radiative capture reaction $^{45}$V(p,γ)$^{46}$Cr, which is identified to impact the $^{44}$Ti nucleosynthesis in Core Collapse Supernovae at astrophysical energies, we performed a spectroscopic study of $^{46}$Cr through the β-decay of the progenitor $^{46}$Mn. We present new results on the decay scheme of the proton-rich exotic nucleus $^{46}$Mn based on a new approach at the level of the experimental data analysis. $^{46}$Mn was produced among other nuclei at the LISE3 fragment separator operated in GANIL using the fragmentation technique. The novel approach is compared with a traditional data analysis, and validated using the known decay scheme of $^{45}$Cr. The observed level scheme of the nucleus $^{46}$Cr is compared with previous works. This work confirms former experimental results, while presenting better statistics and revealing new transitions in the decay scheme.

## Introduction

Presently, the field of astrophysics comprises several aspects related to properties of the atomic nucleus. Among the most fundamental questions are the origin of the elements, the properties of compact objects (neutron stars, white dwarfs), compact binary systems (novae, X-ray bursts, mergers), and stellar collapses and explosions (supernovae) [1–3]. In many of these scenarios, the radioactive decay properties of the nuclei formed in key processes such as the rapid neutron capture process (“r process”) are indispensable. However, in many cases, the most important nuclear data is the thermonuclear reaction rate in the characteristic temperature range of the relevant process. Direct measurements in current radioactive beam facilities of data linked to radioactive decay properties or reaction rates are nowadays impossible for the vast majority of the nuclear species of interest. This statement has to be modulated to some extent, as recent GSI, FRIB, and TRIUMF experiments measured γ-rays and weak r-process related reaction cross sections at energies very close to the Gamow window [4–6]. There are limiting technical factors, such as the low production rate of many of the radioactive species involved. In the case of radiative proton capture—(p,γ) reactions—cross-sections at the relevant energy range in the aforementioned stellar scenarios are below the microbarn, due to the Coulomb barrier.

In isotopes with A ≥ 20 near the stability valley, (p,γ) reactions typically have very high reaction Q values, Q ≥ 5 MeV. Under these conditions, the reaction rates can theoretically be obtained by means of the Hauser-Feshbach statistical model, given the high density of states in the compound nucleus at these excitation energies. In the case of a low density of levels, the statistical model is no longer valid. This occurs especially in very proton-rich nuclei near the proton drip-line, where the radiative proton captures have a low Q value, the reaction being dominated by a limited number of narrow and isolated resonances, plus a much less significant direct contribution. These resonances originate in the existence of excited states of the compound nucleus near the proton emission threshold, where the density of levels is not yet high. In this context, the spectroscopy of radioactive nuclei at energies around the proton emission threshold is revealed as an indirect experimental approach for estimating the thermonuclear reaction rate.

β decay has been widely used since the second half of the twentieth century in order to perform spectroscopic studies of the daughter nucleus [7, 8]. If the Q value of the β decay of the precursor is high enough, states of the daughter nucleus that are above the proton emission threshold may be populated, decaying then by the emission of a proton, known as β-delayed proton emission. Studies in this direction have allowed for the identification of numerous resonances (obtaining energy and restricting the parity and spin of the populated states in the proton emitter), many of which have an impact on the nucleosynthesis occurring in explosive stellar events (see the recent work [9]).

In the nuclear astrophysics context, it is known that the production of $^{44}$Ti occurs during core-collapse supernovae (CCSN) [3, 10, 11], and the reaction $^{45}$V(p,γ)$^{46}$Cr plays a key role in linking the mass region $A \approx 44{-}45$ to heavier nuclei [12–14]. It acts as an escape route, transporting matter from the region where $^{44}$Ti is synthesized to higher masses. If the reaction rate is high, the matter is rapidly converted into heavier species, reducing $^{44}$Ti yields. If it is low, the matter remains trapped and can be reused for $^{44}$Ti production. This reaction also competes with the $\beta ^+$ decay of $^{45}$V, which can recycle material back into $^{44}$Ti. It thus defines a branching point between the destruction and survival of $^{44}$Ti. Uncertainties associated with this reaction directly affect the $^{44}$Ti yields predicted in supernova models.

In this work, we experimentally investigate the β-decay of $^{46}$Mn (progenitor) pursuing two objectives: (i) the spectroscopic study of $^{46}$Cr; (ii) the search for resonant contributions to the reaction $^{45}$V(p,γ)$^{46}$Cr by means of beta-delayed proton emission. There is no experimental information about the radiative capture reaction $^{45}$V(p,γ)$^{46}$Cr, and the existence of narrow resonances that would dominate the cross-section is an open question due to presently unknown excited states in $^{46}$Cr. The experimental setup used for the spectroscopic study allowed for the detection of protons and γ-rays, which makes the identification of excited states in $^{46}$Cr by means of γ-γ and p-γ coincidences possible. In this work, a new data analysis technique regarding p-γ coincidences is presented and compared with a traditional one.

## Experiment

Although the description of the experiment can be found elsewhere [15], some important details are given in the following.

**Fig. 1:**
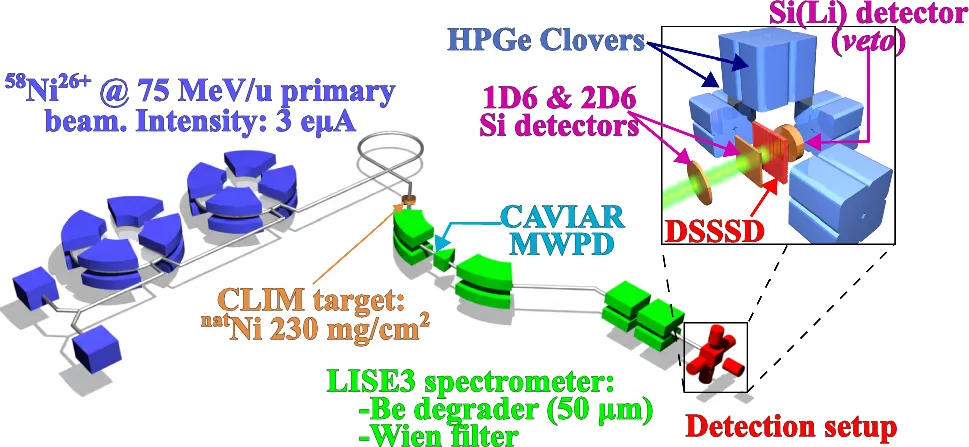
Sketch of the accelerator line for producing the fragmentation beam, the LISE3 fragment separator at GANIL, and the detector setup at the end

The experiment was performed at the LISE3 fragment separator operated in the laboratory GANIL (Caen, France). A $^{58}\hbox {Ni}^{26+}$ primary beam at 75 MeV/u and 3 eμA intensity impinged on a 230 mg/$\hbox {cm}^2$ $^\text {nat}$Ni production target. The resultant fragmentation beam was then purified by means of the fragment separator LISE3, that was adjusted to optimize the selection of $^{48}$Fe, although other kinematically similar isotopes like $^{46}$Mn were not removed from the beam. A multi-wire proportional detector with 96 wires at a 15 mbar isobutene pressure, CAVIAR, was placed in the dispersive plane of LISE3 [16], and its time signal was used for the ToF technique. The detection setup consisted of silicon and silicon-lithium—noted as Si(Li)—charged particles detectors, and a high purity germanium detector array for γ-ray detection, namely HPGe clover detectors (see Fig. 1).

The main set of silicon detectors was composed by two pad detectors, 300 μm and 297 μm thick (referred as 1D6 and 2D6 in Fig. 1), a double-sided silicon strip detector (DSSSD), 316 μm thick and segmented in 16 strips on either side, where the ions of interest were implanted. A 5 mm thick Si(Li) completed the setup.

## Methodology

We have selected $^{46}$Mn among other species in the cocktail beam to study the excited states of its daughter nucleus $^{46}$Cr. As part of our results, we present the proton and γ emission peaks related to the $^{46}$Mn decay and we compare them with previous results presented in references [17, 18]. Also, we present a novel technique in analyzing β-delayed charged particle emission data to study charged particle-γ and γ-γ coincidences. The new methodology has been tested based on the existing experimental information on the $\beta ^+$-decay scheme of $^{45}$Cr. We found new links between γ peaks and the corresponding excited states of $^{45}$V from which they are emitted. Furthermore, using this methodology, compared to results presented in [17], we identify a larger number of proton transitions from the Isobaric Analogue State (IAS) of $^{46}$Cr to $^{45}$V states.

**Fig. 2:**
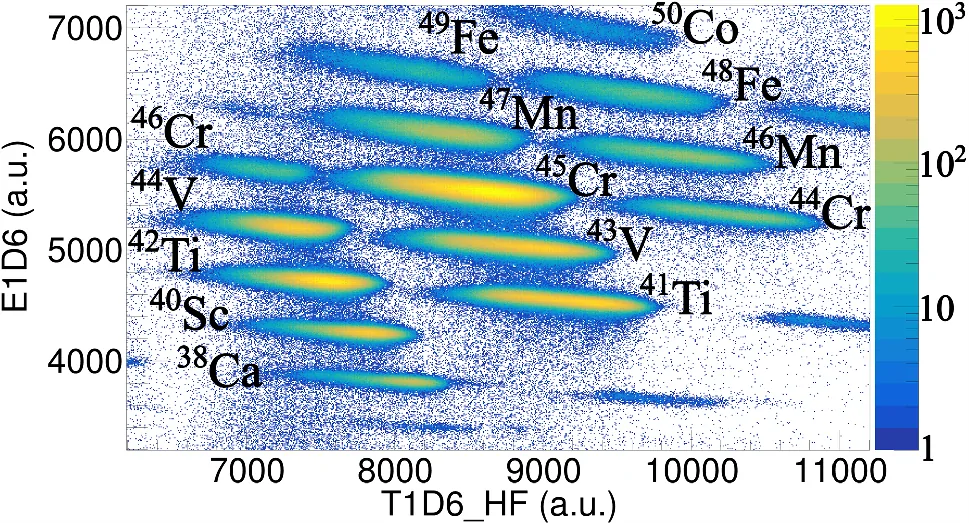
ΔE-ToF technique with Si detector labeled 1D6. The time-of-flight signal was created by means of the time difference between the 1D6 silicon detector and the radiofrequency of the cyclotrons

### Particle identification

Particle identification (PID) of the implanted ions in the DSSSD was made using the time of flight (ToF)-ΔE technique. The energy loss in silicon detector 1D6 was used, together with the time difference between the trigger output due to the particle passing through the corresponding silicon detector and the RF signal of the cyclotron (see Fig. 2). Figure 2 shows the variety of species that are part of the cocktail beam during the optimization of the settings in LISE3, with a prominent intensity for the reference isotope $^{45}$Cr as a consequence of being less exotic than $^{44}$Cr. The isotopes $^{46}$Mn and $^{49}$Fe, as well as the pure β-emitters $^{42}$Ti and $^{46}$Cr can also be identified in the figure. We established graphical selection windows for each isotope identified in Fig. 2. A second graphical selection window for each isotope, now using the time difference between the 1D6 trigger output and the CAVIAR detector, was used to refine the ion selection.

### Correlation of implantation events and decay events

The measurement was performed under the continuous mode operation, as the beam delivered in LISE3 was not implanted in a beam-on/beam-off mode. Thus, implantations in the DSSSD detector and decays of implanted ions were continuously registered by the data acquisition system (DAQ). The implantation and the decay events were sorted out by means of the set of triggers registered by the DAQ. Events with only a trigger signal from the DSSSD detector but no trigger from the detectors 1D6 and CAVIAR upstream in the beam line were classified as decay events. Otherwise, the event is associated to an implantation. The last charged particle detector in the beam line, the so-called Si(Li), worked as a veto allowing for the rejection of events produced by high-energy ions that punched through the DSSSD and were detected in the Si(Li).

**Fig. 3:**
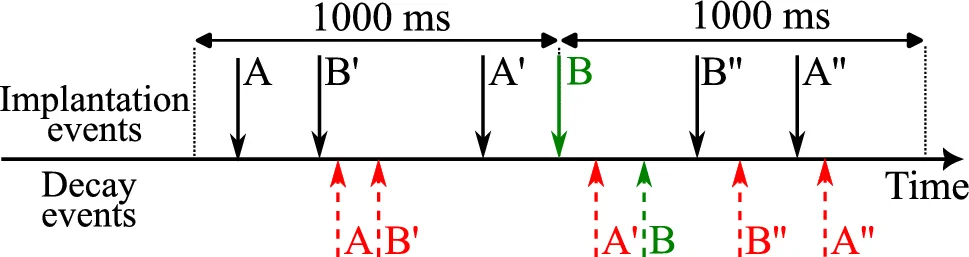
Time correlation scheme. A time window $T_{\text {w}} = 1000$ ms is opened before and after the implantation time of isotope B, located in the center. Within the full-time window of 2000 ms, implantations and decays of isotopes other than B take place, either of a different type of nuclide (A, A’ or A”) or the same type of nuclide (B’, B”)

After selecting a particular isotope by using graphical cuts in the ToF versus ΔE spectra, as for the isotope $^{46}$Mn, the energy spectra of the emitted particles associated with its decay were obtained by means of spatial and time correlations. The method was already applied in [17]. For the time correlations, a time window of several times its half-life $T_{\text {w}}=1$ s ($T_{\text {w}}=2$ s in the case of $^{45}$Cr) was opened before and after one implantation event, as shown in Fig. 3. Then, a search for decay-type events that occurred during that time window was performed. For the spatial correlation, as the DSSSD vertical and horizontal strips form a grid of pixels, among all the decay events, only those firing the pixel where the implantation occurred, were selected.

**Fig. 4:**
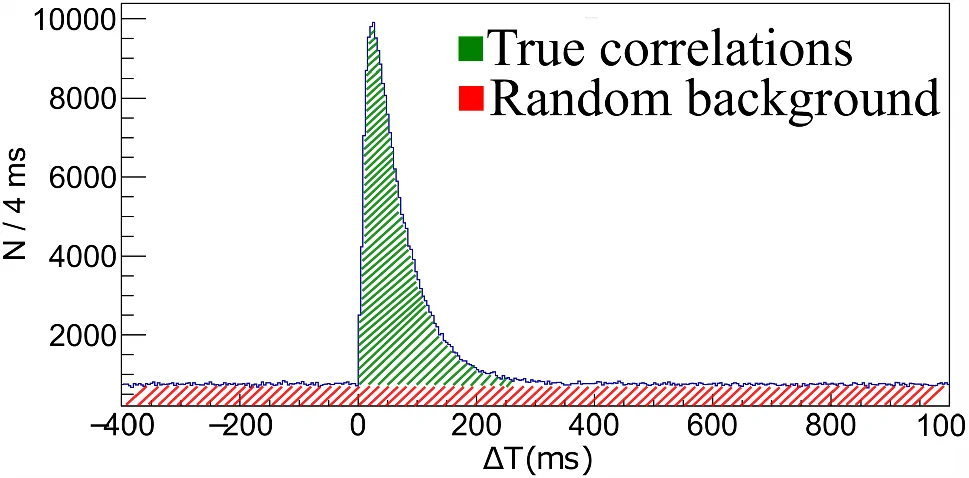
Decay-time spectrum of $^{46}$Mn in the present work. The background level is represented in red, while the signal is represented in green

While taking the time difference between the implantation $T_{\text {imp}}$ and decay events $T_{\text {dec}}$, $\Delta T =T_{\text {dec}}-T_{\text {imp}}$, the negative values (ΔT<0) are only associated with random coincidences (background). In Fig. 4 the resulting decay-time spectrum of $^{46}$Mn is shown, where the background level is represented in red while the signal is in green, following the same color code used in Fig. 3. Energy spectra (of protons or γ-rays) for events at negative times, ΔT<-5 ms, are subtracted from energy spectra built with events at positive times, ΔT>5 ms. In order to use this method, we only considered isotopes implanted in the central strips of the DSSSD, and with their implantation time satisfying the condition $T_{\text {w}}< T_{\text {imp}} < T_{\text {run}}-T_{\text {w}}$, used also in [19], which accounts for the opening and closing of each data file on tape, where $T_{\text {run}}$ stands for the time duration of the measurement (the clock for the time stamp is reset to zero when closing a data file) the value of which depends on each run (typical values of $T_{\text {run}}\approx 1$ h and 13 min).

**Fig. 5:**
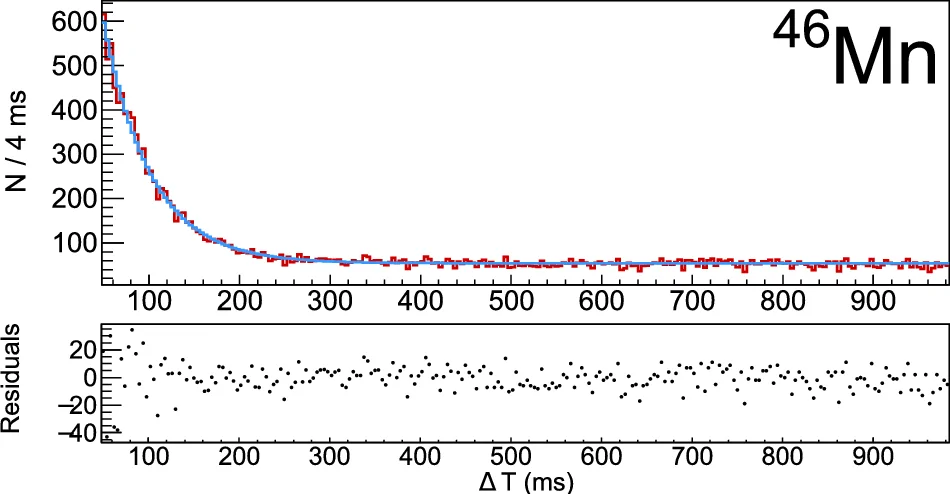
Top panel: Decay-time spectrum for the $^{46}$Mn isotope obtained in the present work gated in the proton peak 3 of Fig. 13, $2.25~\text {MeV}<E<2.5~\text {MeV}$, the so-called energy cut. The solid line in blue color reflects the fit used to infer the half-life, $\hbox {T}_{1/2} = 36.3(4)$ ms, with a $\chi ^2/D.o.F.=0.96$. Bottom panel: The fit’s residuals are shown

From the decay-time spectrum in the top panel of Fig. 5, obtained using the energy cut $2.25~\text {MeV}<E<2.5~\text {MeV}$, we can derive the half-life of the isotope $^{46}$Mn. Exponential fits were used plus a constant to account for the background. The fit gives a half-life value of $\hbox {T}_{1/2} = 36.3(4)$ ms, statistical error only, with a $\chi ^2/D.o.F.=0.96$. The bottom panel of the figure shows the fit residuals. The result is in good agreement with the value $\hbox {T}_{1/2} = 36.2(4)$ ms in [17].

### Calibration of the detectors

The 32 strips of the DSSSD detector were initially calibrated using a triple α-source ($^{239}$Pu, $^{241}$Am, and $^{244}$Cm). In a second iteration, the calibration was refined using well-known β-delayed proton emission peaks of $^{49}$Fe, $^{45}$Cr, $^{41}$Ti, and $^{50}$Co, as components of the cocktail beam.

The ${\beta }$ detection efficiency of the DSSSD strips was obtained by selecting pure β emitters within the cocktail beam as $^{46}$Cr and $^{42}$Ti. In such cases, only the number of implanted isotopes $N_{\text {imp}}$, and the number of β particles, $N_\beta$, are needed [19]:

1 $$\begin{aligned} \epsilon _\beta = \frac{ N_\beta }{N_{\text {imp}} \left( 1-\tau \right) }. \end{aligned}$$

**Fig. 6:**
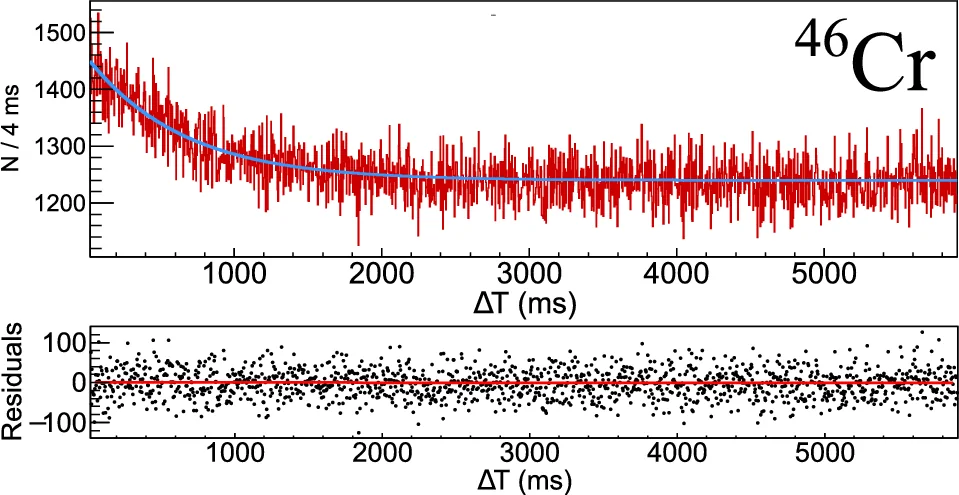
Top panel: Decay-time spectrum for the $^{46}$Cr isotope obtained in the present work. The solid line in blue color reflects the fit used to infer the half-life, which follows the Bateman’s equation which contain an exponential, for the $^{46}$Cr decay, a curve for the grow-in and decay of the daughter $^{46}$V, and a constant to account for the background. The fitted value was the $^{46}$Cr half-life, while the $^{46}$V one was taken from the literature [20]. $\hbox {T}_{1/2} = 224.4(8)$ ms, with a $\chi ^2/D.o.F.=1.04$. Bottom panel: the fit’s residuals are shown, the red line marks a zero-degree polynomial fit

τ is the dead time of the DAQ, with values around 11%. τ is obtained using two counters counting a 100 Hz pulse generator during the data acquisition, but one of them was inhibited by the dead time when the DAQ recorded an event. The ratio of both counters results in the live time of the DAQ, the complement of the dead time τ, which is a feature of the data acquisition. $N_{\text {imp}}$ is known from the isotope sorting, while $N_\beta$ is obtained from the fit applied to the decay-time spectrum (see Fig. 6 for the $^{46}$Cr isotope). In the case of $^{46}$Cr, only the half-life of the nucleus $^{46}$V, $\hbox {T}_{1/2} = 422.62(5)$ ms, was considered for the fit, as $^{46}$Ti is stable [20]. Similarly, in the case of $^{42}$Ti, only the half-life of the nucleus $^{42}$Sc, $\hbox {T}_{1/2} = 680.79(28)$ ms, was considered for the fit, as $^{42}$Ca is stable [20].

The value for the half-life of $^{46}$Cr derived in this work is $\hbox {T}_{1/2} = 224.4(8)$ ms, with a $\chi ^2/D.o.F.=1.04$, in good agreement with the value from [17], $\hbox {T}_{1/2} = 224.3(13)$ ms. Additionally, the half-life of $^{42}$Ti derived in this work is $\hbox {T}_{1/2} = 212.1(10)$ ms, with a $\chi ^2/D.o.F.=1.03$, which is in reasonable agreement with the value of $\hbox {T}_{1/2} = 208.14(45)$ ms reported in [21], and with the value of $\hbox {T}_{1/2} = 211.7(19)$ ms reported in [22]. Taking into account the above considerations, we get a value for the β-detection efficiency of $\epsilon _\beta =$ 14.5(3)%. The proton detection efficiency is $\epsilon _p= 1$ for energies of 100 keV $<E_p$. For energies $E_p>4$ MeV, the protons deposit only part of their energy in the DSSSD detector, therefore, their energy value is not complete.

In the case of the four HPGe clover detectors, we used $^{56}$Co, $^{60}$Co, $^{207}$Bi, and $^{133}$Ba+$^{137}$Cs sources to calibrate the 16 crystals and compute the γ detection efficiency using the following equation:

2 $$\begin{aligned} \epsilon _\gamma = \frac{ N_\gamma \text {Div} }{I_\gamma A T_{\text {run}} \left( 1-\tau \right) }, \end{aligned}$$

where $N_\gamma$ is the area of the peak for a given γ photon, Div is the divisor value set in the trigger rate for the γ detectors, $I_\gamma$ is the branching ratio, *A* the activity of the source, $T_{\text {run}}$ the duration of the calibration run, and τ the dead time of the data acquisition. Those calibration points were later used to fit the curve $f(E) = \exp {\left( a+b\log {\left( E \right) } \right) }$ to obtain the γ-ray detection efficiency at any given energy. The values obtained for the fit were a=6.78(2) and b=-0.669(2).

## Novel p-γ coincidence analysis technique

One of the methods to study p-γ coincidences is through energy windows. Usually, the focus of the study is on charged particle energy spectra, obtained by gating on a particular γ-ray peak.

We propose an alternative technique to identify p-γ coincidences. We applied an increasing energy threshold in the DSSSD energy spectrum, $E_{\text {cut}}$, in steps of 50 keV. For each value of $E_{\text {cut}}$, we looked into the particle-γ spectrum, and integrated the counts in each $\gamma _i$ peak separately, ${N_\gamma }_i$, following its variation as $E_{\text {cut}}$ is increasing. For a given $\gamma _i$ line, its intensity for the processes $\beta -p-\gamma$ (β-decay followed by the emission of a proton and a γ-particle) and $\beta - \gamma$ is given in [19] as:

3 $$\begin{aligned} I_{\beta , p, \gamma }{}_i= &   \frac{ N_\gamma {}_i}{\epsilon _p \epsilon _\gamma N_{\text {imp}} \left( 1-\tau \right) },\nonumber \\ I_{\beta , \gamma }{}_i= &   \frac{ N_\gamma {}_i }{\epsilon _\beta \epsilon _\gamma N_{\text {imp}} \left( 1-\tau \right) }, \end{aligned}$$

where ${N_\gamma }_i$ is the number of counts in the $\gamma _i$ peak, $N_{\text {imp}}$ the number of implantations for the selected isotope, τ the dead time of the DAQ, $\epsilon _\gamma$ the γ detection efficiency, $\epsilon _\beta$ the beta detection efficiency and $\epsilon _p$ the proton detection efficiency. These intensities will vary as the applied $E_{\text {cut}}$ increases, but we can notice that ${N_\gamma }_i$ is the only quantity affected by this. It is easier to see this, if we normalize the intensity to the initial value ${I_\gamma }_{i0}$ (i.e., $E_{\text {cut}} = 0$). Let us define the normalized intensity ${{\mathcal {I}}_\gamma }_i(E_{\text {cut}})$ as follows:

4 $$\begin{aligned} {{\mathcal {I}}_\gamma }_i(E_{\text {cut}}) \equiv \frac{{I_\gamma }_i (E_{\text {cut}})}{{I_\gamma }_{i0}}. \end{aligned}$$

This definition will apply for either $\beta -p-\gamma$ or $\beta - \gamma$ processes, only changing $\epsilon _p$ for $\epsilon _\beta$ accordingly. Then, without loss of generality, we can see that ${{\mathcal {I}}_\gamma }_i(E_{\text {cut}})$ reduces to the expression:

5 $$\begin{aligned} {{\mathcal {I}}_\gamma }_i(E_{\text {cut}}) = \frac{\frac{ {N_\gamma }_i (E_{\text {cut}})}{\epsilon _\beta \epsilon _\gamma N_{{\text {imp}}} \left( 1-\tau \right) }}{\frac{ {N_\gamma }_{i0} }{\epsilon _\beta \epsilon _\gamma N_{\text {imp}} \left( 1-\tau \right) }} = \frac{ {N_\gamma }_i (E_{\text {cut}})}{{N_\gamma }_{i0} }. \end{aligned}$$

Therefore, variations of ${{\mathcal {I}}_\gamma }_i(E_{\text {cut}})$ for the $\gamma _i$ peak are intimately linked with the spectrum of charged particles. In a plot of the curve ${{\mathcal {I}}_\gamma }_i(E_{\text {cut}})$ versus $E_{\text {cut}}$, a sizable decrease within a range of $E_{\text {cut}}$ values will reflect a correlation in that region of the charged particle spectrum, meaning that charged particles in that range of $E_{\text {cut}}$ are in coincidence with the $\gamma _i$ gamma line. Otherwise, no significant change will mean there is no correlation at all in that part of the charged particle spectrum. As ${{\mathcal {I}}_\gamma }_i(E_{\text {cut}})$ is a monotonically decreasing function of $E_{\text {cut}}$, it is expected for its derivative to be negative or null, ${{\mathcal {I}}}'_\gamma (E_{\text {cut}})$ ≤0. Therefore, in the proposed method to study p-γ coincidences, we will work with the negative of the derivative. To simplify the notation, we will write the numerical derivative as:

6 $$\begin{aligned} {\mathcal {I}}'_\gamma (E_{\text {cut}}) \equiv \frac{d {\mathcal {I}}_\gamma (E_{\text {cut}})}{dE_{\text {cut}} }. \end{aligned}$$

In order to validate this new method, we have applied it to the isotope $^{45}$Cr, whose beta-decay scheme is already partially known.

### The technique applied to $^{45}$Cr

**Fig. 7:**
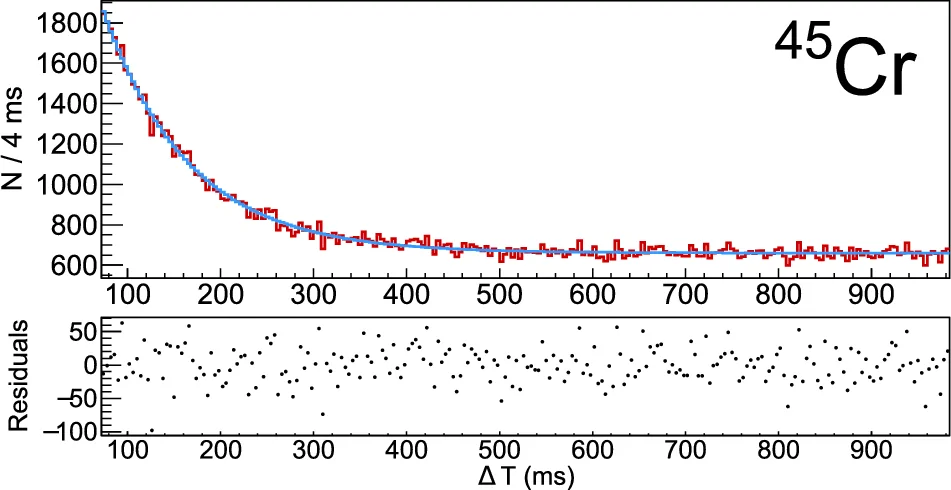
Top panel: Decay-time spectrum for the $^{45}$Cr isotope obtained in the present work gated in the new proton peak of Fig. 8, $1.8~\text {MeV}<E<2.0~\text {MeV}$. The solid line in blue color reflects the fit used to infer the half-life, $\hbox {T}_{1/2} = 64.3(7)$ ms, with a $\chi ^2/D.o.F.=0.98$. Bottom panel: The fit’s residuals are shown

Selecting the isotope $^{45}$Cr in our data, we obtained the decay-time spectrum shown in the top panel of Fig. 7, by using an energy cut of $1.8~\text {MeV}<E<2.0~\text {MeV}$. The solid line in blue color reflects the fit used to infer the half-life, $\hbox {T}_{1/2} = 64.3(7)$ ms, statistical error only, with a $\chi ^2/D.o.F.=0.98$. In the bottom panel, the residuals of the fit are shown. The decay of $^{45}$Cr was previously studied by Dossat et al. [17]. The reported value therein for the half-life of $^{45}$Cr is $\hbox {T}_{1/2} = 60.9(4)$ ms. To solve the discrepancy between the half-life values, we repeated the measurement but gating on the γ-rays 1, and 3, which correspond to those with the highest intensities. We obtained similar values to our half-life: $\hbox {T}_{1/2} = 63.3(5)$ ms with a $\chi ^2/D.o.F.=1.03$ for $E_\gamma =1083$ keV, and $\hbox {T}_{1/2} = 64.1(22)$ ms with a $\chi ^2/D.o.F.=0.98$ for $E_\gamma =1370$ keV.

**Fig. 8:**
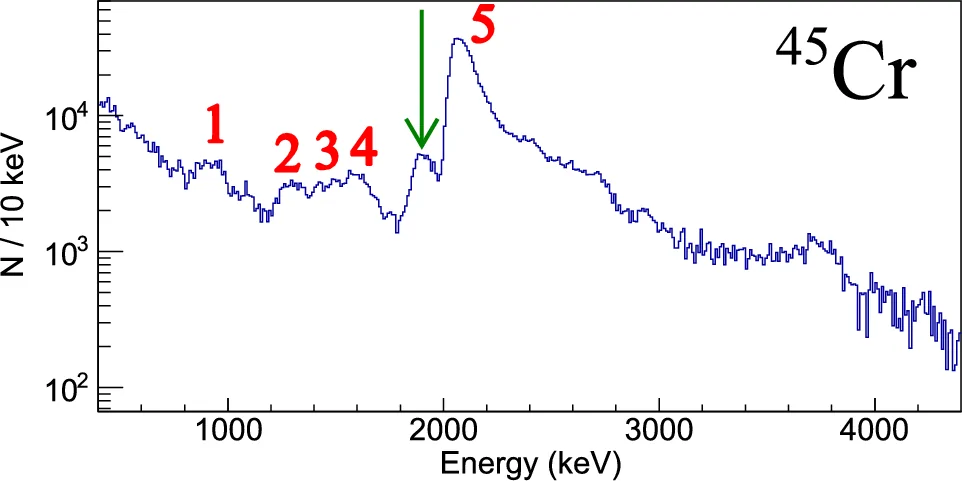
Energy spectrum for the DSSSD strips from 400 keV to 4400 keV for the decay of $^{45}$Cr. The green arrow points to a new proton peak identified in this work. The red numbers indicate the proton peaks reported in [17]

**Fig. 9:**
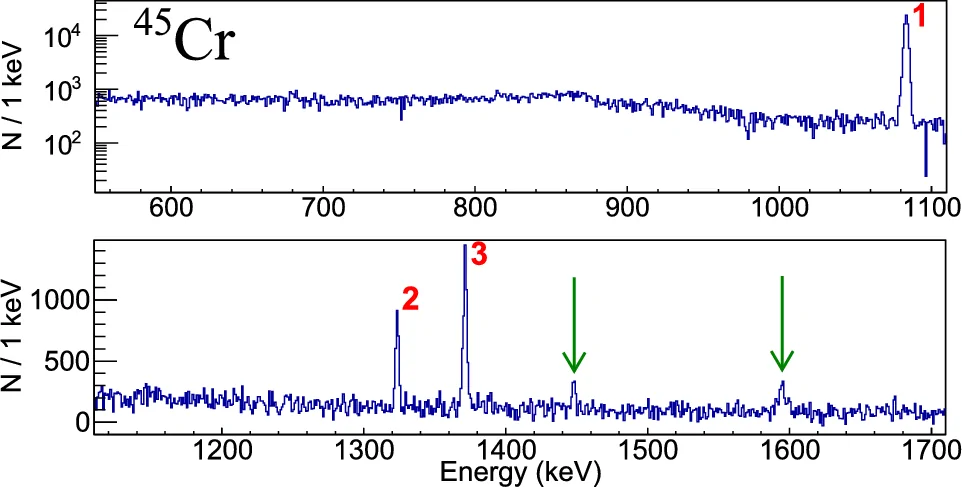
Energy spectrum for the HPGe crystals from 540 keV to 1110 keV, and from 1110 keV to 1710 keV for the decay of $^{45}$Cr. Green arrows point to new γ lines identified in this work. The red numbers indicate the previously seen γ-ray peaks by [17]

**Table 1:** Protons peaks and γ lines observed in this work for the $^{45}$Cr $\beta ^+$-decay, shown in Figs. 8, 9. Results include all the peaks reported in [17], labeled with numbers, and the arrows correspond to the new proton and γ-rays found in this work. Proton energies are centre-of-mass energies

Proton		γ-ray	
Label	Energy (keV)	Label	Energy (keV)
1	945(31)	1	1083.3(1)
2	1303(25)	2	1322.7(3)
3	1468(27)	3	1370.0(5)
4	1609(28)	→	1447.5(2)
5	2087(9)	→	1594.7(2)
→	1907(3)		

**Fig. 10:**
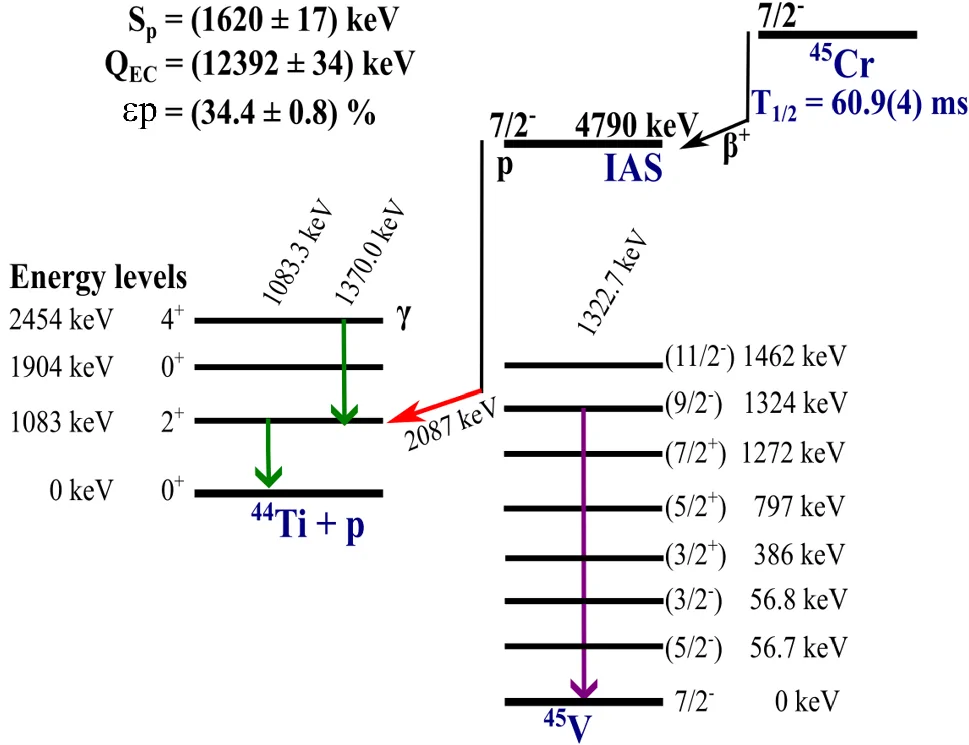
Decay scheme for $^{45}$Cr $\beta ^+$-decay, modified from [17]. The energies of the emitted γ-rays are vertically written. Only the proton emitted from the IAS of $^{45}$V is included. εp is the delayed proton emission probability, not to be confused with the proton detection efficiency $\epsilon _p$. Values are taken from the literature [17]

Based on the decay-time spectrum of $^{45}$Cr, we got the corresponding energy spectrum for charged particles, Fig. 8, and the γ-rays spectrum, Fig. 9. All the peaks reported in [17], related to the $\beta ^+$-decay of $^{45}$Cr, are confirmed in our work. Furthermore, in the charged particle spectrum resulting from our data, we found a structure around 1900 keV (marked with a green arrow), not reported in [17]. In addition, two new γ lines can also be found in the photon energy spectrum around 1450 keV and 1590 keV (also marked with green arrows). These new structures will not be discussed here, but in more detail in an upcoming work. The known decay scheme for $^{45}$Cr is shown in Fig. 10. The green arrows depict the γ lines corresponding to $^{44}$Ti de-excitation, while the purple arrow shows the γ-peak corresponding to $^{45}$V de-excitation. The proton transition from the $^{45}$V IAS to the $^{44}$Ti excited state is presented with a red arrow. The γ cascade in the $^{44}$Ti de-excitation, $4^+ \rightarrow 2^+ \rightarrow 0^+$, shown in the decay scheme in Fig. 10, has been confirmed by our analysis as the 1083 keV and 1370 keV γ-rays were detected in coincidence.

**Fig. 11:**
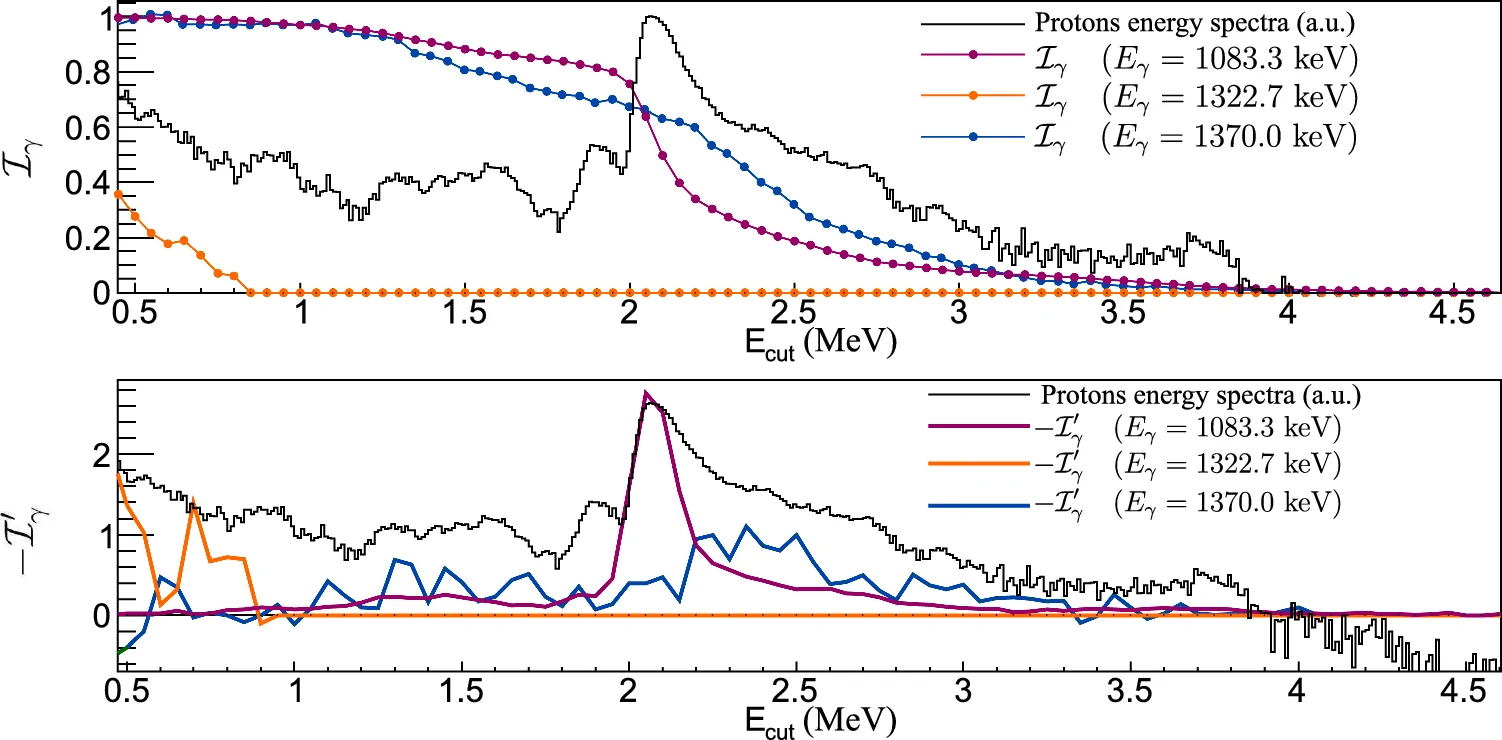
Top panel: Normalized intensity curves ${{\mathcal {I}}_\gamma }_i(E_{\text {cut}})$ for the γ lines $E_\gamma = 1083.3, 1322.7, 1370.0$ keV linked to the $^{45}$Cr decay; Bottom panel: $-{{\mathcal {I}}'_\gamma }_i(E_{\text {cut}})$ for the same γ lines. The charged particle energy spectrum, black spectrum, is overlaid to follow where the threshold, $E_{\text {cut}}$, is affecting

Applying our method to study p-γ coincidences to the γ lines found in this work, the curves shown in Fig. 11 are obtained. In the top panel, we show ${\mathcal {I}}_\gamma$ as $E_{\text {cut}}$ increases in steps of 50 keV. It must be emphasized that ${\mathcal {I}}_\gamma$ is independent of efficiencies and dead time as it is a quotient, hence all those quantities being cancelled out. In the bottom panel, the derivative curves, $-{\mathcal {I}}_\gamma '(E_{\text {cut}})$, for the different γ-rays are shown with lines. In both panels, we keep the charged particle energy spectrum related to the decay of $^{45}$Cr as a background figure in black serving as a visual reference. We can see that ${\mathcal {I}}_\gamma$, corresponding to the $E_\gamma = 1322.7$ keV ($^{45}$V: $9/2^- \rightarrow 7/2^-$ (g.s.) de-excitation), vanishes at $E_{\text {cut}} \approx 1$ MeV. In addition, $-{\mathcal {I}}_\gamma '$ marks a broad peak in that region. The latter marks the coincidence of this γ line with the β region of the energy spectrum, which demonstrates it is a $\beta -\gamma$ process.

**Fig. 12:**
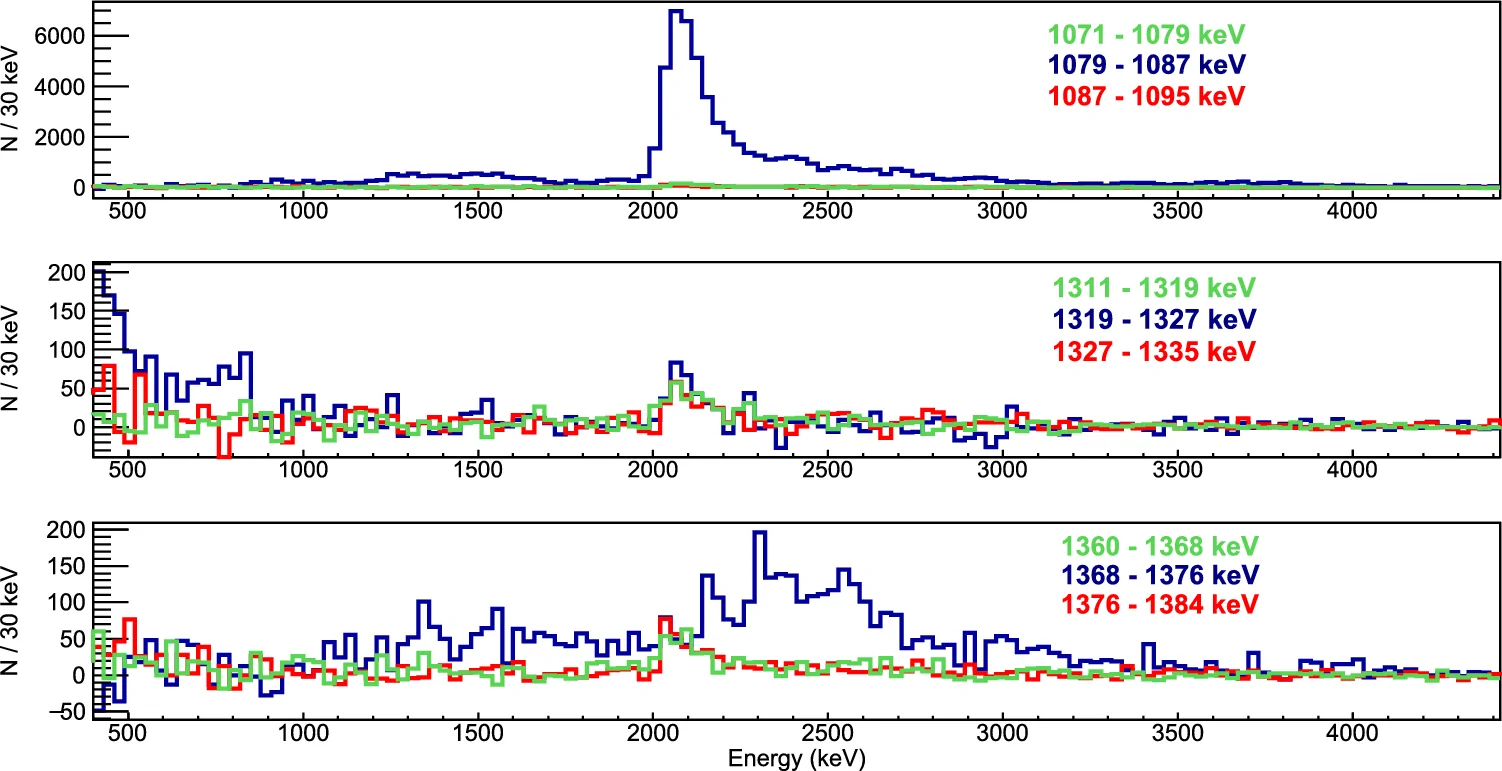
$^{45}$Cr DSSSD spectra gated on: top the 1083 keV γ line; middle the 1322.7 keV γ line; bottom the 1370.0 keV γ line. The blue curve is generated with a gate on the γ-ray energy, while the green and the red curves are gated on a region below and above the γ-ray peak, respectively

In contrast, for ${\mathcal {I}}_\gamma$ corresponding to the $E_\gamma = 1083.3$ keV ($^{44}$Ti: $2^+ \rightarrow 0^+$ de-excitation) and $E_\gamma = 1370.0$ keV ($^{44}$Ti: $4^+ \rightarrow 2^+$ de-excitation), the curve remains almost constant up to $E_{\text {cut}} \approx 1$ MeV. This means that there are no coincidences with the β region, consistent with them being $\beta -p-\gamma$ processes. For both γ lines, there is a notable decrease in ${\mathcal {I}}_\gamma$ in the energy range 2 MeV $<E_{\text {cut}}< 3$ MeV, although it is higher for $E_\gamma = 1083.3$ keV. With the $-{\mathcal {I}}_\gamma '$ curves in the bottom panel, we can identify one prominent peak in the purple curve around $E_{\text {cut}} = 2.1$ MeV. It is interesting to notice that the protons decaying from the IAS of $^{45}$Cr to the $^{44}$ $\hbox {Ti}^{2+}$ excited state carry an energy of $E_p=2.087$ MeV. The blue curve shows two broad structures, one around 1 MeV $<E_{\text {cut}}< 2$ MeV and the second one at 2.2 MeV $<E_{\text {cut}}<3.4$ MeV.

We also studied the p-γ coincidences by means of gating in the γ-ray spectrum and plotting the resulting charged particles energy spectrum, see Fig. 12. The gate was 8 keV wide, significantly larger than the FWHM found for the γ-rays, centered on each γ line studied, and the resulting charged particle spectrum is shown with a blue line. In order to unambiguously associate a γ line with a peak in the proton spectrum, two additional windows were used at lower and higher energies and are shown with red and green lines, respectively. In the top panel, we can see this method applied to the $E_\gamma = 1083.3$ keV, where we can see a main peak in the charged particles spectrum at around $E_{\text {cut}} = 2100$ keV, and two broad structures at 1000 keV <E<1700 keV and 2300 keV <E<3000 keV in the coincidence spectrum. The charged particle spectrum resulting from moving the gate up and down is in the background level. In the middle panel, the case for the $E_\gamma = 1322.7$ keV is shown, where a coincidence is visible around E=2100 keV. Nevertheless, as this structure is also seen when moving the gate up and down, the coincidence is discarded, and we associate these peaks in coincidence with Compton scattered γ-rays of a higher energy γ line. Then, only the coincidence at low energies E<900 keV, in the region of β particles, remains. In the bottom panel, we can see the case for $E_\gamma = 1370.0$ keV, where multiple peak-like structures appear in the coincidence spectrum.

Comparing the results of the two methods, it is confirmed that the new method, i.e., using ${\mathcal {I}}_\gamma$, establishes the same p-γ correlations as the standard method that relies on applying cuts in the γ-ray spectrum. Using ${\mathcal {I}}_\gamma$, we can even distinguish β-γ from β-p-γ processes by means of the low-energy part of the spectra, where a broad structure emerges in coincidence with the region where the β distribution is located. This is the case of the $E_\gamma =1322.7$ keV γ-ray line, whose $-{\mathcal {I}}_\gamma '$ presents only one broad structure in the derivative curve at the low-energy part of the spectrum. For the β-p-γ cases, like the 1083.3 keV γ line, their main proton peak contributor is well resolved, and it is also possible to see smaller contributors. In addition, the structures found both in the $-{\mathcal {I}}_\gamma '$ and the energy cut curves at E≥200 keV could suggest transitions coming from $^{45}$V excited states different than the IAS.

## The new findings for the $^{46}$Mn $\beta ^+$-decay

**Fig. 13:**
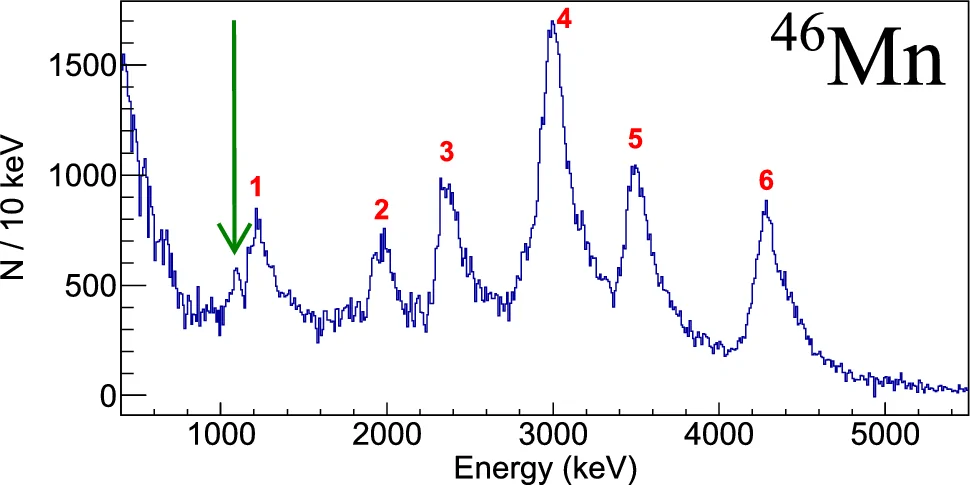
Charged particle energy spectrum for the decay of $^{46}$Mn measured in the present work (see Table 2 for number labels). The green arrow points to a proton peak newly identified in this work. The red numbers indicate the previously seen protons in [17]

**Fig. 14:**
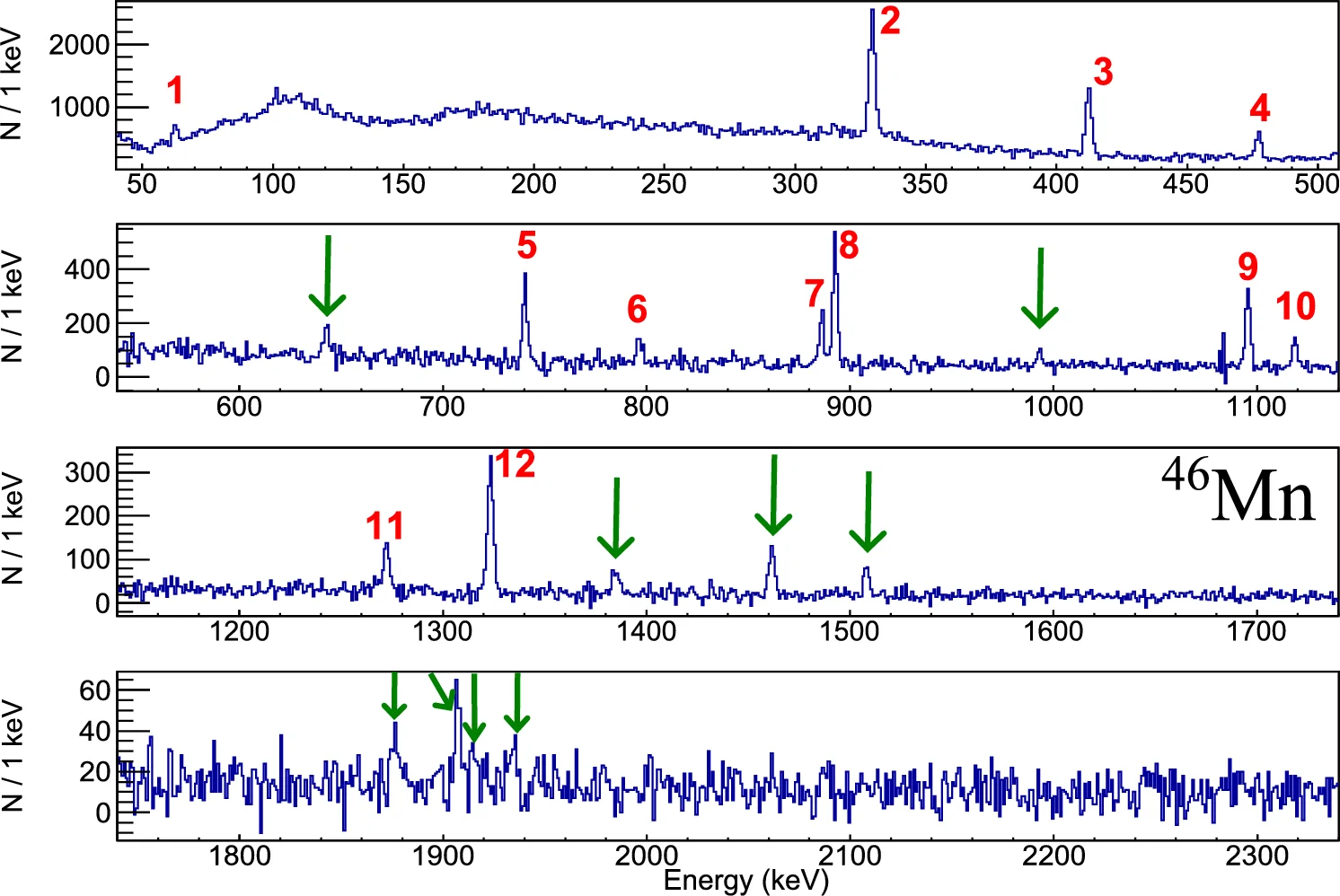
Energy spectrum for γ-rays emitted in the decay of $^{46}$Mn in the present work: from 40 keV to 508 keV (top), from 540 keV to 1140 keV (second), from 1140 keV to 1740 keV (third), and from 1740 keV to 2340 keV (bottom) (see Tables 2, 3). The range from 508 keV to 540 keV is avoided due to the high statistics of the 511 keV annihilation line. Green arrows point to new γ lines identified in this work. The red numbers indicate the previously seen γ-ray peaks by [17]

The $^{46}$Mn isotope was amongst the outgoing fragments from the production target that reached the detection setup. The number of implantations of the $^{46}$Mn isotope into the DSSSD was high (438,460 implantations during 109 h and 38 min of data acquisition), making it possible to get a reasonably good statistics. This can be seen in the improvement of the energy spectra of Figs. 13, 14 with respect to those previously obtained in [17, 18].

### Energy spectra of charged particles and γ-rays

The charged particle spectrum observed in the DSSSD detector for the decay of $^{46}$Mn, including the full statistics and after background subtraction, is shown in Fig. 13. In Fig. 14 we show the γ-ray spectrum divided into four energy regions of all the data collected in the set of 16 HPGe crystals in coincidence with $^{46}$Mn.

**Table 2:** Same as Table 1 for the $^{46}$Mn. Known proton and γ-ray lines for the $^{46}$Mn $\beta ^+$-decay reported by Dossat et al. in Tab 11 of [17], labeled with numbers. The arrows correspond to the new proton and γ-rays found in this work

Proton		γ-ray	
Label (keV)	Energy	Label	Energy (keV)
1	1224(12)	1	54.4(5)
2	2090(20)	2	329.4(2)
3	2358(13)	3	410.2(2)
4	3003(13)	4	475.2(3)
5	3494(25)	5	739.7(7)
6	4254(15)	6	796.1(2)
→	1069(12)	7	885.7(7)
→	2800(12)	8	892.5(3)
→	2945(11)	9	1094.7(4)
		10	1118.0(15)
		11	1272.6(5)
		12	1322.1(5)
		→	644.0(2)
		→	993.1(2)
		→	1385.3(2)
		→	1462.0(2)
		→	1506.9(2)
		→	1877.3(2)
		→	1907.3(2)
		→	1917.2(2)
		→	1933.2(2)

In the low energy part of the spectrum in Fig. 13, (E<1000 keV), one can find the typical tail caused by the β particles. For energies above 1000 keV, seven structures are clearly seen. Most of these structures, labeled with numbers from 1 to 6 (see Table 2), correspond to proton peaks reported in [17]. It is worth mentioning that all the peaks observed in that work are present in our results, but the new spectra have better resolution and higher statistics. Besides, the LISE3 settings in the experimental measurement resulted in a larger $^{46}$Mn production than in the aforementioned works, the settings of which were centered on $^{45}$Fe$/^{48}$Ni. Furthermore, we found a new proton peak at 1069(12) keV, not previously seen. Nevertheless, we still face the fact that the charged particle energy spectrum is affected by the pile-up produced by the high counting rate of β particles. These events of β particles hitting the pixel where a proton is detected worsen the detector resolution. As a result, this pile-up causes a tail on the right side of each proton peak.

The γ-ray spectra obtained from the decay of $^{46}$Mn, Fig. 14, recover all the γ lines found in [17] and shown in Table 2. Furthermore, in the present work, we find five γ lines not previously reported, marked with arrows in the figure and presented in Table 3.

**Table 3:** γ-ray lines in $^{46}$Mn $\beta ^+$-decay reported or assigned for the first time in this work (green arrows in Fig. 14)

Energy (keV)	Associated process
644.0(2)	$^{45}$V$^{9/2+}$ to $^{45}$V$^{7/2+}$
993.1(2)	Not identified
1385.3(2)	Not identified
1462.0(2)	$^{45}$V$^{11/2-}$ to $^{45}$V$^{7/2-}$ g.s.
1506.9(2)	$^{46}$Cr$^{*}$ to $^{46}$Cr$^{4+}$
1877.3(2)	Not identified
1907.3(2)	Not identified
1917.2(2)	$^{45}$V$^{9/2+}$ to $^{45}$V$^{7/2-}$ g.s.
1933.3(2)	Not identified

**Fig. 15:**
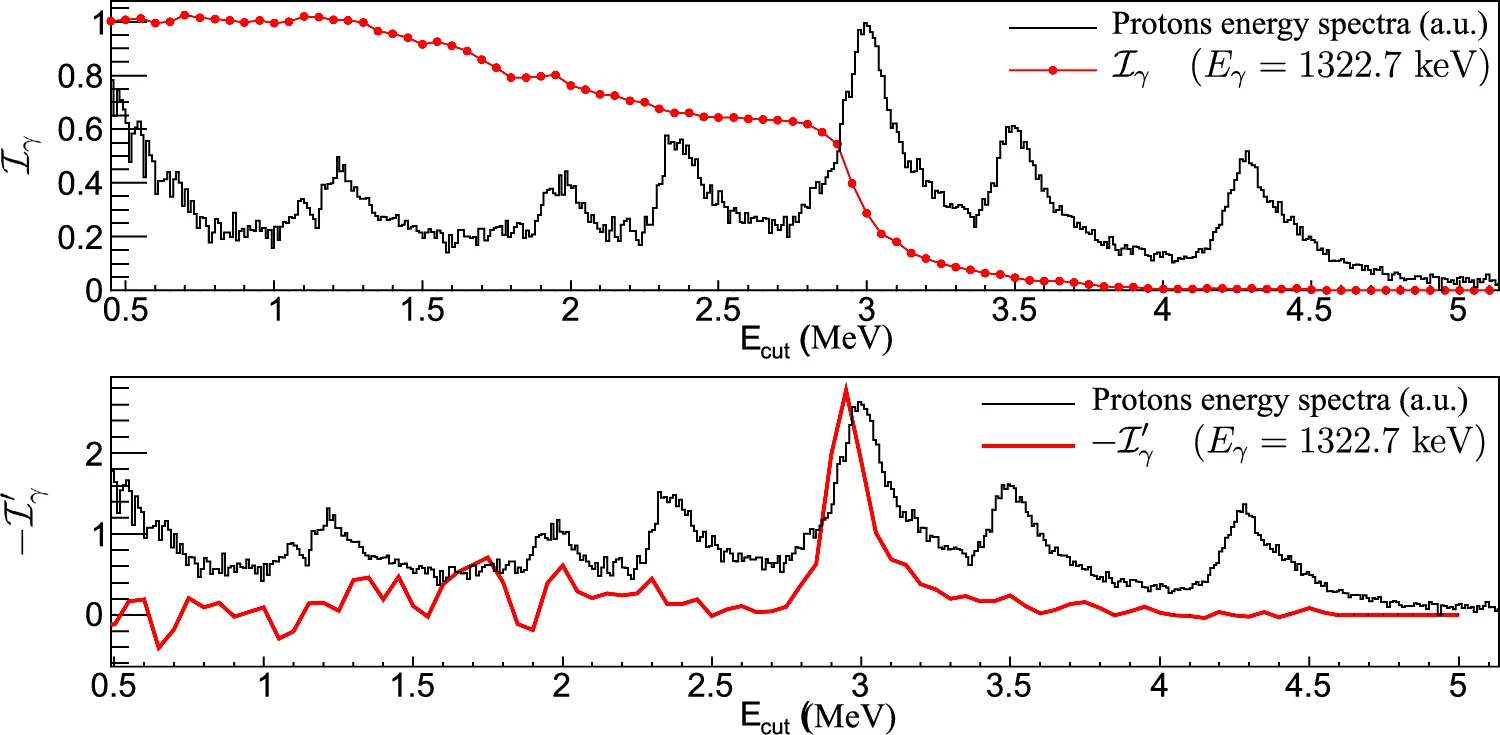
Top panel: Normalized intensity curves ${{\mathcal {I}}_\gamma }_i(E_{\text {cut}})$ for the γ-ray line $E_\gamma = 1322.7$ keV linked to the $^{46}$Mn decay; Bottom panel: $-{{\mathcal {I}}'_\gamma }_i(E_{\text {cut}})$ for the same γ-ray line. The charged particle energy spectrum, black spectrum, is overlaid to follow where the threshold, $E_{\text {cut}}$, is affecting

### p-γ and $\gamma -\gamma$ coincidences

**Fig. 16:**
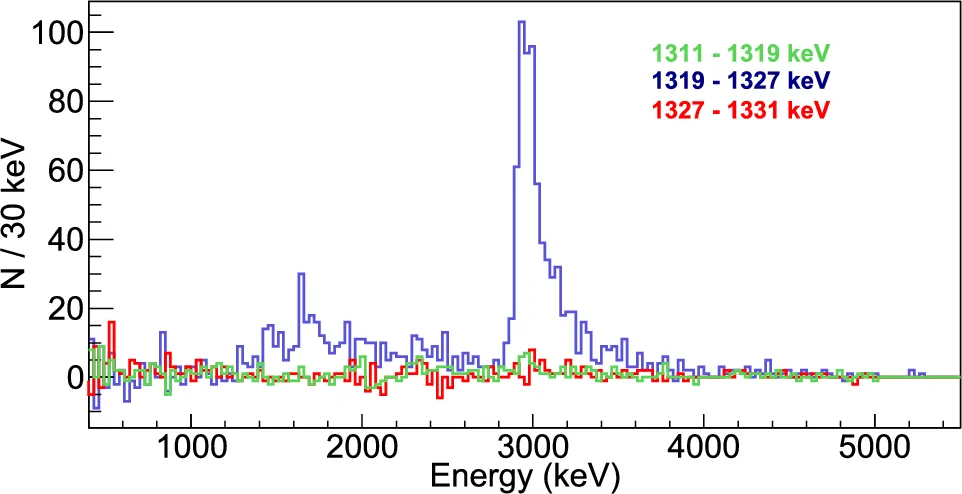
The $^{46}$Mn DSSSD spectra gated on the $E_\gamma =1322.7$ keV γ-ray line is depicted in blue color. The green and red curves correspond to spectra gated below and above the peak of interest

A comparison between the two aforementioned methods has been made for the 1322.7 keV γ-ray emitted in the decay of $^{46}$Mn. The results are shown in Figs. 15, 16, for the method based on ${{\mathcal {I}}_\gamma }(E_{\text {cut}})$ and that using selection cuts, respectively. In the top panel of Fig. 15, we show ${\mathcal {I}}_\gamma$ as $E_{\text {cut}}$ increases in steps of 50 keV; while in the bottom panel, the derivative curve, $-{\mathcal {I}}_\gamma '(E_{\text {cut}})$, for the 1322.7 keV γ-ray is shown with the red line. In both panels we keep the charged particle energy spectrum related to the decay of $^{46}$Mn as background, black line, serving as a visual reference. Opposite to the $^{45}$Cr $\beta ^+$-decay where the ${\mathcal {I}}_\gamma$ vanishes at $E_{\text {cut}} \approx 1$ MeV; for the $^{46}$Mn $\beta ^+$-decay, ${\mathcal {I}}_\gamma$ vanishes around $E_p\approx 3.5$ MeV. Furthermore, $-{\mathcal {I}}_\gamma '(E_{\text {cut}})$ marks a clear coincidence peak at energies around $E_p\approx 3$ MeV. In addition, $-{\mathcal {I}}_\gamma '$ marks some smaller peak-alike structures at lower energies. All of the above is in agreement with this γ-ray being part of a $\beta -p-\gamma$ process for the $^{46}$Mn $\beta ^+$-decay.

The blue spectrum of Fig. 16 is the energy spectrum of charged particles detected by the DSSSD detector after applying a selection centered on the 1322.7 keV γ line. Two additional energy spectra are obtained when the selection cut is displaced to lower and to higher energies than that of the peak of interest (green and red color spectra, respectively). The two latter spectra are at the background level, hence the coincidence between the 1322.7 keV γ line and the 3 MeV peak in the charged particle energy spectrum becomes evident. What is also interesting to notice is that there might be some coincidences around energies of $1600~\text {keV}< E<1800$ keV (see Fig. 16), this will be analysed in a future work.

**Table 4:** $\gamma -\gamma$ and γ-p coincidences seen in this work for the $^{46}$Mn $\beta ^+$-decay. The * symbol stands for coincidences with less than 5σ above background

γ (keV)	γ-rays in coincidence (keV)	Protons in coincidence (keV)
329.4	410.2, 475.2, 644.0, 885.7, 1118.0	2358, 3006, 3494
410.2	329.4, 475.2, 1118.0	2358, 3006, 3494
475.2	329.4, 410.2, 739.7, 796.1*	2358, 3006
644.0	329.4*, 885.7	2358
739.7	410.2*, 475.2*	2358, 3006, 3494
796.1	–	3494
885.7	329.4, 644.0	3006
892.5	1094.7	β region
993.1	–	β region
1094.7	892.5	β region
1118.0	329.4, 410.2	2358
1272.6	–	3006
1322.7	–	2945
1385.3	–	2945*
1462.0	–	2800
1506.9	892.5, 1094.7	β region
1877.3	–	2358
1907.3	–	β region
1917.2	–	2358
1933.3	–	2358

We carried out p-γ and $\gamma -\gamma$ coincidence analyses in the observed set of γ-rays. The results are collected in Table 4, and we describe them in what follows.

**Fig. 17:**
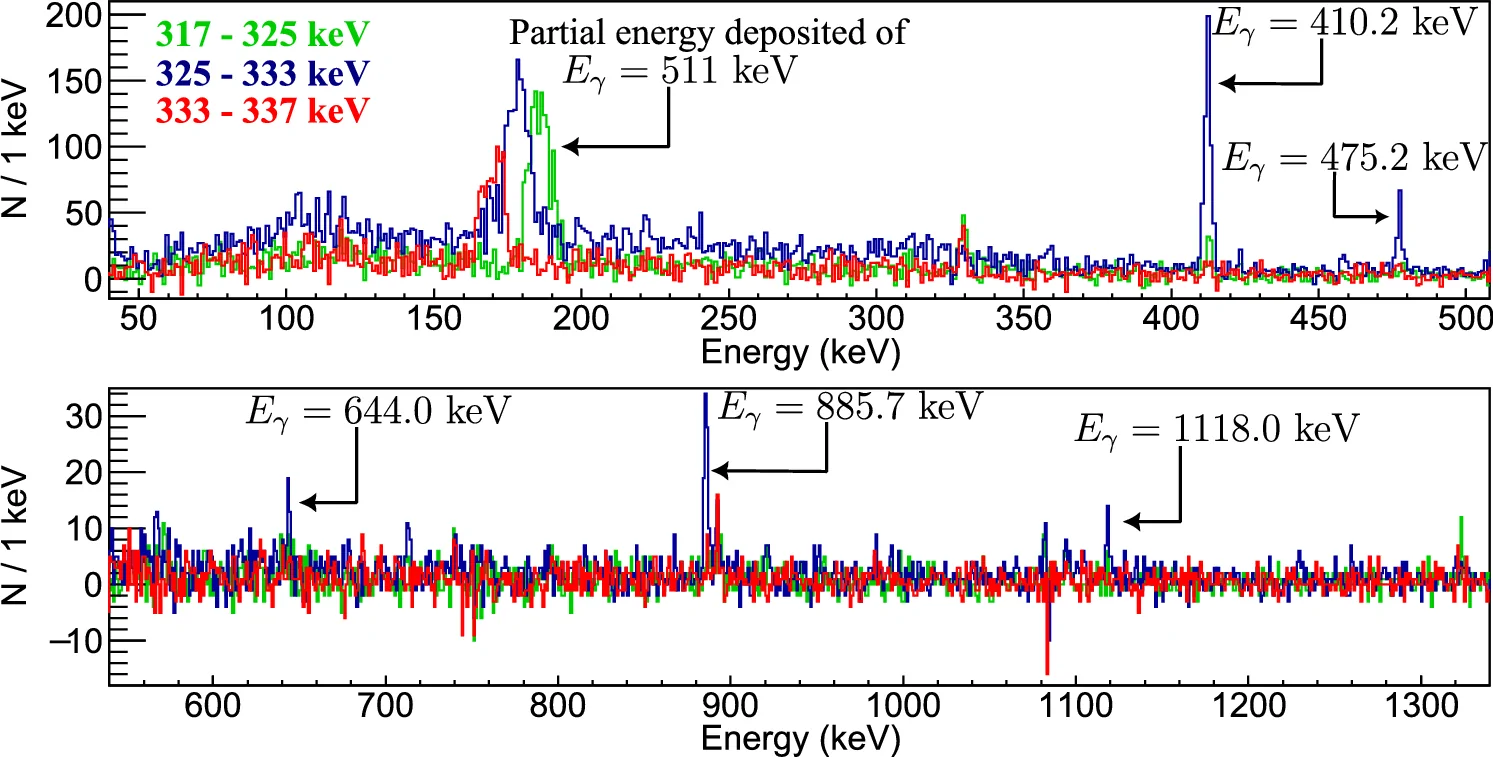
The $^{46}$Mn HPGe clover detectors summed spectrum gated on the 329.4 keV γ-ray line is depicted in blue color. The γ coincidences are highlighted with black arrows, including the partial energy deposition of the 511 keV annihilation peak. The red and the green spectra are obtained from shifting the gate above and below the peak of interest

**Fig. 18:**
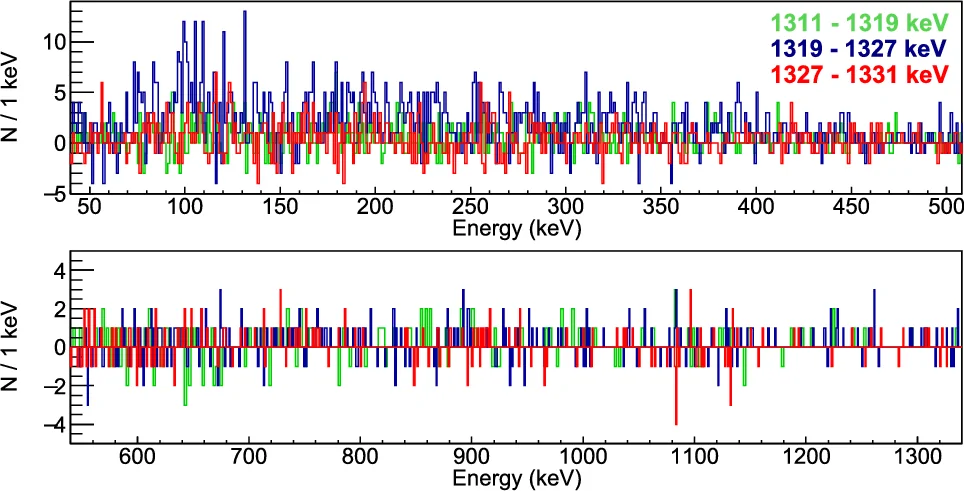
Same as Fig. 17, but for the γ-ray at $E_\gamma =1322.7$ keV

The blue spectrum of Fig. 17 is the energy spectrum of γ-rays detected by the HPGe clover detectors after applying a selection window centered on the 329.4 keV γ line. Two additional energy spectra are obtained when the selection window is displaced to lower and to higher energies than that of the peak of interest (green and red color spectra, respectively). The two latter spectra are at the background level, hence there is an apparent coincidence between the 329.4 keV γ line and the following 5 γ lines in the γ-ray energy spectrum: 410.2 keV, 475.2 keV, 644.0 keV, 885.7 keV, and 1118.0 keV. The coincidence with the peak around E=175 keV comes from a partial energy deposition of the $E_\gamma =511$ keV. This is due to the Compton scattering in one of the crystals, alongside the Compton-scattered γ-ray observed in another crystal. Another example is given in Fig. 18, where a selection window centered on the 1322.7 keV γ line is set. There, no clear association can be made with any other γ-ray.

We can see in Table 4 that the $E_\gamma = 1322.7$ keV and $E_\gamma =1462.0$ keV do not have any coincidences with other γ rays, while the $E_\gamma = 475.2, 885.7, 1272.6$ keV γ-rays have coincidences with γ-rays different from those of this set. The latter is in agreement with the fact that these γ rays de-excite the same $^{45}$V excited state, $7/2^+$, to different final states ($5/2^+$, $3/2^+$, and $7/2^-$, respectively). In addition, $E_\gamma =1462.0$ keV seems compatible with the $11/2^-\rightarrow 7/2^-$ de-excitation found also in [20]. Furthermore, from Tables 2, 4 we see that all these γ-rays are in coincidence with the 3 MeV structure in the energy spectrum of charged particles, Fig. 13. The energy conservation results in the following form:

7 $$\begin{aligned} E_{x}(^{46}\text {Cr}^*)&= E_{x}(^{45}\text {V}^*) + E_p + S_p(^{46}\text {Cr}), \end{aligned}$$

where $S_p(^{46}\text {Cr}) = 4883$ keV is the proton separation energy of $^{46}$Cr, $E_p$ is the energy of the emitted proton (centre-of-mass), and $E_{x}(^{45}\text {V}^*)$ and $E_{x}(^{46}\text {Cr}^*)$ are the energies of the excited states of $^{45}$V and $^{46}$Cr, respectively.

If we assume the structure around 3000 keV in the spectrum of charged particles to be originated by protons of one single energy at 3006 keV (new energy determined in this work), emitted from the IAS of $^{46}$Cr, the energy of the excited state in $^{45}$V should be 1263 keV, which does not match the energies of $^{45}\hbox {V}^{9/2-}$, and $^{45}\hbox {V}^{11/2-}$. Another hypothesis is proposed: excited states of $^{45}$V, ${9/2^-}$, and ${11/2^-}$ to be populated from the IAS of $^{46}$Cr by emission of protons of different energies. To test this, we chose to deepen our study into the proton structure located at 3 MeV.

**Fig. 19:**
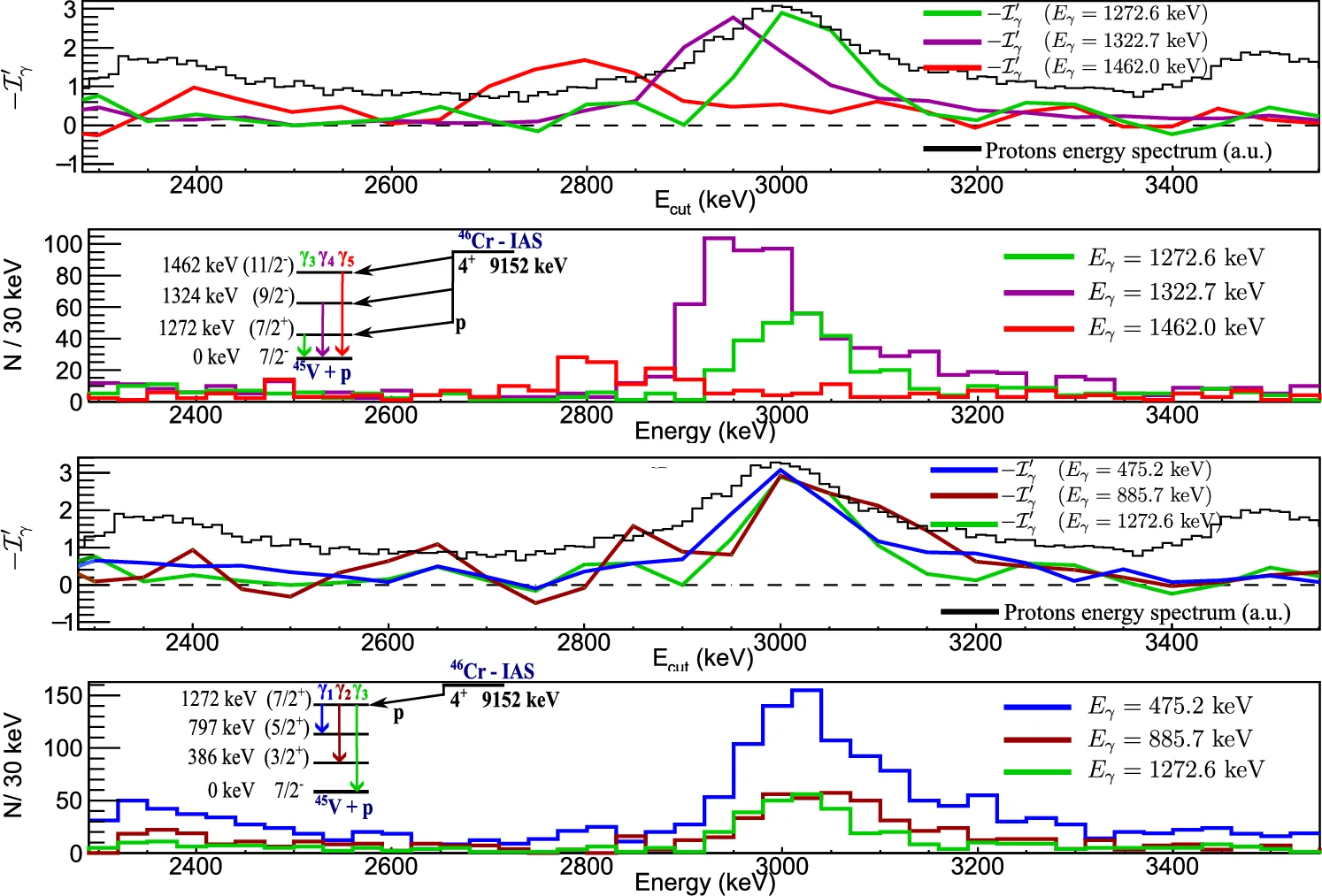
Comparison of energy cut and derivative techniques for $^{46}$Mn γ-rays in coincidence with the 3 MeV structure in the energy spectrum of charged particles. Top: The $-{\mathcal {I}}_\gamma '$ curves for the 1272.6 keV, 1322.7 keV, and 1462.0 keV γ-rays are shown, where it can be seen that the peaks around 3 MeV are not aligned. Middle-up: Charged particle spectra after gating on the same γ-rays. Centroids of the peaks coincide with those observed in the $-{\mathcal {I}}_\gamma '$ curves. Middle-down: The $-{\mathcal {I}}_\gamma '$ curves for the 475.2 keV, 885.7 keV, and 1272.6 keV γ-rays are shown. Bottom: Charged particle spectra after gating on the γ-rays 475.2 keV, 885.7 keV, and 1272.6 keV. In both cases, it can be seen that the proton peaks do align. The respective partial decay scheme is shown, not to scale

Particularly, we focused in those γ-rays which have the largest coincidence in the DSSSD spectrum around the 3 MeV structure, and with no other γ coincidence: $E_\gamma = 1272.6,$ 1322.7, 1462.0 keV. We applied our technique to these γ-rays, as seen in the top of Fig. 19, where we can appreciate that the centroids of the different proton peaks do not align. Two peaks are clearly shifted to lower energies. This is also seen with the energy cut technique depicted in the middle panel of Fig. 19. In contrast, the centroids of the proton peaks do align if we choose other γ-rays that are also in coincidence with the proton emission around $E_p=3003$ keV previously assigned by [17], (in this work, computed as $E_p=3006(10)$ keV), which de-excite the same level in $^{45}$V, the γ-rays at $E_\gamma = 475.2, 885.7, 1272.6$ keV. This is shown on the bottom panel of Fig. 19, where we can see that the proton peaks align at the same energy. What we can conclude from the above is that the 3 MeV structure is in reality caused by, at least, three unresolved proton peaks.

The energies of the proton peak centroids were obtained from fits applied to the gated charged particle energy spectra for different γ-rays. In the case of the γ-rays at $E_\gamma =1322.7, 1462.0$ keV, we only used one gated energy spectrum to obtain the energy of the protons of $E_p=2945(11)$, 2800(12) keV, respectively. For the third proton peak we used an average of the fitted spectra gated on the $E_\gamma =475.2,$ 885.7, 1272.6 keV, resulting in a proton of $E_p=3006(10)$ keV. The above energies of the protons and their coincidence with the respective γ-rays, which coincide with the de-excitation energies of $^{45}$V from the $7/2^+$, $9/2^-$, and $11/2^-$ states, are compatible with proton emissions from the $^{46}$Cr IAS to those final states of $^{45}$V.

**Fig. 20:**
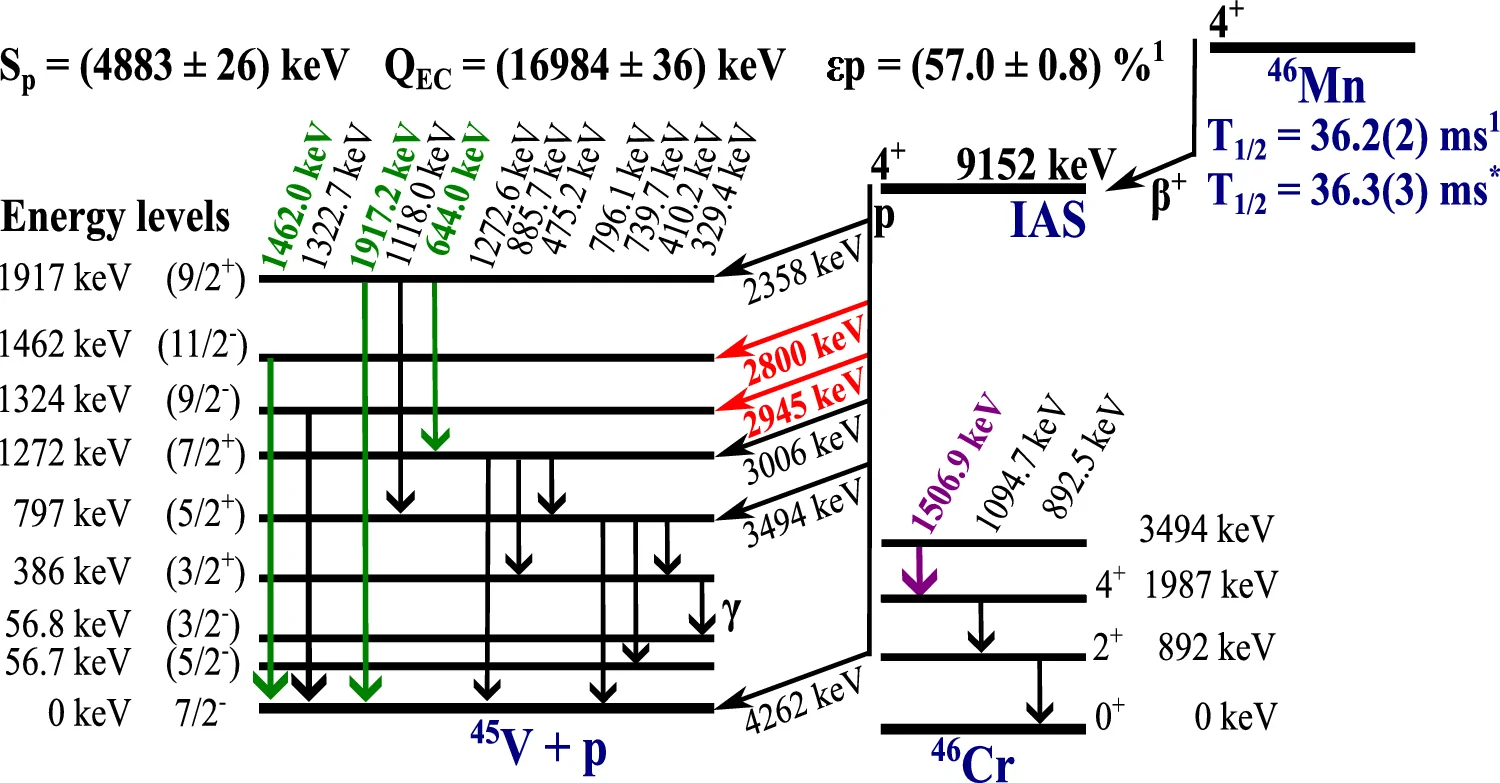
Decay scheme for the $^{46}$Mn $\beta ^+$-decay. The green arrows depict the new γ-rays found in this work compatible with $^{45}$V de-excitation, while with a purple arrow we show the γ-ray peak compatible with $^{46}$Cr de-excitation. As well, the red arrows represent the new proton transitions found in this work. (1) Values taken from [17]; (*) Weighted mean value obtained from this work and [17]

We could confirm the population of the excited state in $^{46}$Cr at 3494.3 keV by the $\beta ^+$-decay of $^{46}$Mn. The $-{\mathcal {I}}_\gamma '$ curve for the 1506.9 keV γ-ray ends at the low-energy part of the spectrum, so there is only one broad structure in the derivative curve that points to a β-γ process. Additionally, we explored other γ-rays in coincidence with the one at 1506.9 keV. The results are shown in Table 4. This γ-ray is compatible with the transition of $^{46}$Cr from the level at 3494.3 keV to the $4^+$ state at 1987 keV [20]. This new feature is included in the decay scheme in Fig. 20.

### New $\beta ^+$-decay scheme

New transitions, either in the form of γ-ray emission, or proton emission followed by the emission of a γ-ray could be identified in this work. The results are summarized in Table 4 and Fig. 20. In the decay scheme, the newly identified γ-ray transitions in $^{45}$V are depicted with green arrows, while those corresponding to $^{46}$Cr are in purple. Proton emission identified in this work from the $^{46}$Cr IAS to excited states of $^{45}$V are represented by arrows in red.

## Summary and conclusions

In this work, part of the data collected in the experiment E666, carried out at the LISE3 fragment separator of the GANIL accelerator laboratory, is analysed. The analysis focuses mainly on data related to the decay of $^{46}$Mn, although, in order to validate the newly proposed technique, data corresponding to the decay of $^{45}$Cr are also examined. The data exhibit significantly more statistics than those obtained in previously published experiments. The setup used made it possible to obtain the time-decay curves of both nuclides, as well as to detect γ-rays (up to  2 MeV) and the protons emitted following the $\beta ^+$ decay. The experimental half-lives we obtain for both nuclides show a reasonable agreement with previously published values. The combined detection of protons and γ-rays enabled a detailed study of p-γ and $\gamma -\gamma$ coincidences following the $\beta ^+$-decay. The work presents a new technique for analyzing p-γ coincidences. In this technique, tracking the variation of the intensity curve of a given γ-ray as the minimum energy, $E_{\text {cut}}$, of the charged particles detected in coincidence with that γ-ray, $\gamma _i$, is increased, allows one to establish charged particle-γ coincidences. The technique is particularly useful for discerning whether the emitted γ-ray originates from the daughter nucleus ($\beta -\gamma$ coincidence) or from the residual nucleus after proton emission (β-p -γ coincidence). The results thus obtained for the decays of $^{46}$Mn and $^{45}$Cr are compared with those provided by the traditional technique employing selection windows in the γ-ray spectrum. A scrutiny of the results using both analysis methods for the case of $^{45}$Cr allows the validation of the new technique. Proton-emission lines and γ-ray lines previously unknown in the decay of $^{45}$Cr are presented ($E_{\gamma } = 1447.5, 1594.7$ keV, $E_p = 1907$ keV), although the identification of the initial and final states remains pending for future work. The γ-ray spectrum of the $^{46}$Mn decay obtained in the experiment analysed in this work reveals nine previously unknown γ-ray lines (see Table 3). The analysis of p-γ coincidences has allowed us to identify proton emissions with energies $E_p = 2800(12), 2945(11), 3006(10)$ keV, which had not been reported before. Altogether, the new experimental results shown in the present work obtained from the E666 experiment so far contribute to enriching the decay scheme of $^{46}$Mn. The present work deepens into the level scheme of $^{46}$Cr and consequently its nuclear structure. Regarding the $^{45}$V(p,γ)$^{46}$Cr reaction rate, the new data do not appear to have an impact on it, as we have not found any evidence of a new excited state of $^{46}$Cr that could influence it. It is well known that an experimental approach employing a gas detector like a time projection chamber instead of silicon detectors would allow the detection of protons with energies below 1 MeV. Combined with a γ-ray detection system of the TAS (Total Absorption Spectrometer) type, which is particularly efficient for identifying cascade γ-rays, such an approach would provide a more detailed understanding of the processes accompanying the β-delayed proton emission and the nuclear structure of the daughter and residual nuclei. Although the physics case in this work lies in the field of nuclear astrophysics, the novel technique proves to be a complementary powerful tool for studying p-γ coincidences in β-delayed proton emission experiments.

## Data Availability

Data will be made available on reasonable request. [Author’s comment: The datasets generated and/or analysed during the current study are available from the corresponding author on reasonable request.]

## Code Availability Statement

Code/software will be made available on reasonable request. [Author’s comment: The code/software generated and/or analysed during the current study is available from the corresponding author on reasonable request.]
